# Tracking Microbial Evolution in the Subseafloor Biosphere

**DOI:** 10.1128/mSystems.00731-21

**Published:** 2021-08-17

**Authors:** Rika E. Anderson

**Affiliations:** a Department of Biology, Carleton College, Northfield, Minnesota, USA

**Keywords:** marine subsurface, microbial evolution

## Abstract

The deep marine subsurface constitutes a massive biosphere that hosts a multitude of archaea, bacteria, and viruses across a diversity of habitats. These microbes play key roles in mediating global biogeochemical cycles, and the marine subsurface is thought to have been among the earliest habitats for life on Earth. Yet we have a poor understanding of what forces govern the evolution of subsurface microbes over time. Here, I outline why evolutionary trajectories in the subsurface may be different than those of microbes living on the surface of the planet and describe how we can take advantage of technological advancements to study the evolutionary dynamics of subsurface microbes and their viruses. The sequencing revolution, in tandem with marine infrastructure advancements, promises that we will soon gain a much deeper understanding of how the vast majority of the microbial biosphere changes, adapts, and evolves over time.

## COMMENTARY

Approximately one-half of all the carbon, nitrogen, phosphorus, sulfur, and iron on Earth is processed by marine microbes and the viruses that infect them (1–5). About 80% of all archaea and bacteria on Earth inhabit the subsurface, and of those, the majority can be found in the multitude of habitats that make up the marine subsurface (6, 7). These include habitats ranging from highly productive deep-sea hydrothermal vents, to the rocky, porous crust near ridge flanks, to the low-biomass sediments below ocean gyres. While some of these habitats are energy-rich and host abundant, diverse microbial communities, others are severely energy-limited and host as few as 100 cells per square cm (7). These microbes encompass the majority of the biomass in our oceans (6, 7) and thus impose strong controls on global biogeochemical cycles (8).

Nevertheless, little is known about how these microbial populations adapt and evolve over time. Understanding microbial adaptations, stability, and resilience in response to perturbations in the deep sea is critical for understanding the impact of subsurface microbes on marine biogeochemistry. Moreover, these insights can help scientists and stakeholders understand how subsurface microbes may respond to anthropogenic impacts like climate change and deep-sea mining. Unresolved questions include the following: how do marine subsurface microbial and viral communities respond to environmental changes? What are the strongest drivers of microbial and viral ecology and evolution in the subsurface over time? How does this vary depending on the habitat? Here, I will discuss some of the ways in which we might expect microbial evolution to operate differently in the marine subsurface and outline some steps that we can take to answer some unresolved questions.

## WHY MIGHT MICROBIAL EVOLUTION BE DIFFERENT IN THE SUBSURFACE?

**FIG 1:**
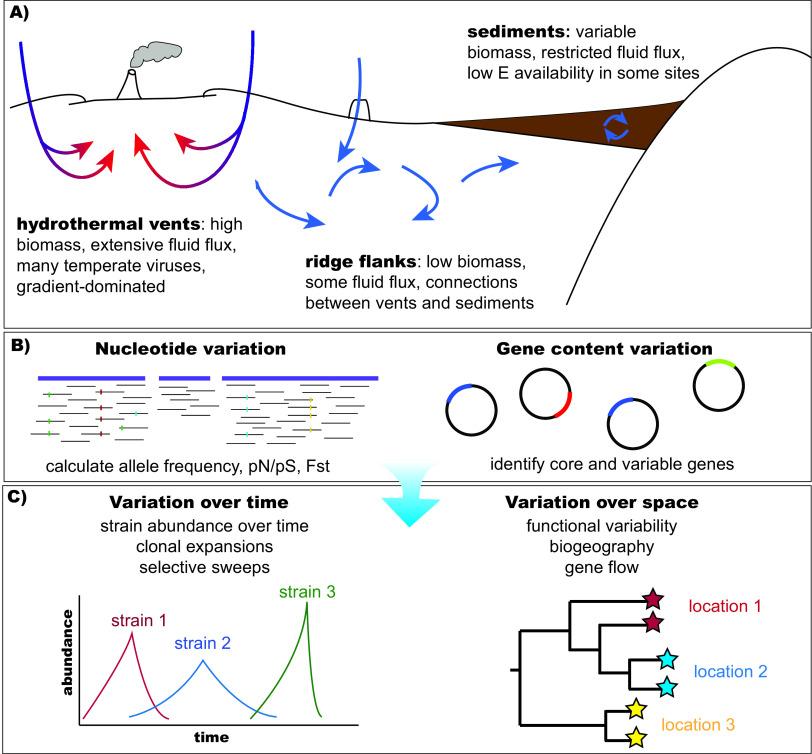
Schematic diagram of different habitats in the marine subsurface (A), methods we can use to track genomic variants (B), and scales over which evolutionary dynamics can be observed (C).

The principles guiding microbial evolution likely vary dramatically depending on the subsurface habitat, particularly depending on energy availability, fluid flux, and microbial biomass and population size (9) (Fig. 1A). Microbial cells buried in marine sediments, especially in oligotrophic regions such as below the South Pacific Gyre, are extremely energy-limited (10), restricting abundances to approximately 100 to 1,000 cells/cm^3^ (7). Canonical studies of microbial evolution have traditionally focused on lab-based strains with high cell counts (e.g., reference 37) and/or natural microbial lineages that are in high abundance in their habitats (e.g., references 38 to 40). Thus, these foundational studies derive conclusions primarily from microbial populations that are sufficiently large to be governed by natural selection. However, small populations are more susceptible to genetic drift, leading to the hypothesis that in severely energy-limited environments with low biomass, evolution is guided not by the forces of natural selection but instead by largely stochastic processes. Some of our work in energy-restricted habitats has borne this out (11), but more research is necessary. These ideas lead to questions about how an ecosystem governed largely by stochastic forces evolves and changes over long timescales.

Moreover, populations in energy-starved habitats have extremely low replication rates and are likely to persist in stationary phase, raising the question of how rapidly evolution occurs in such communities. Microbial growth rates in the marine subsurface, particularly in low-energy habitats, are not well constrained (12, 13), with some estimates of generation times in the range of thousands of years (14). Furthermore, while some previous work in culture has suggested that nutrient-starved cells exhibit increased mutational frequency (15), *in situ* work has suggested that microbes in deeply buried sediments exhibit low mutation rates and selective survival of taxa (16). Thus, in energy-limited sedimented regions of the subsurface, evolution dominated by random chance and exceedingly low replication rates could facilitate the existence of microbial populations in which deleterious or at least nonadvantageous mutations accumulate, approximating a version of Muller’s ratchet (17) applied across an entire ecosystem. Clearly, more work is needed to resolve these questions.

Drastically different conditions are observed in the subsurface biosphere at deep-sea hydrothermal vents, where energy availability and cell abundances are high. Here, extreme conditions, constant fluid flux, and environmental gradients in temperature, pH, and redox conditions create unusual conditions for natural selection and evolution. These unique attributes include high rates of horizontal gene transfer (18), high cell contact rates through biofilms (e.g., reference 41), and a high incidence of viral lysogeny (19, 20). Previous work by our lab suggests that selection in these habitats is driven largely by geochemistry, nutrient availability, and energy availability (21–23). Viruses are subject to these selective forces as well, acting temporarily as mutualists by carrying auxiliary metabolic genes that facilitate energy transformation in these habitats (24, 25).

Despite their disparate geochemical and physical characteristics, the distinct habitats of the marine subsurface—ranging from marine sediments, to ridge flanks, to hydrothermal vents—are linked by fluid flow, raising the question of how biogeography and gene flow shape subsurface microbial populations. While some of our work suggests that hydrothermal vent microbes and their viruses are fairly dispersal-limited and thus restricted to specific geographic regions (26, 27), others have found surprisingly widespread connectivity across subsurface habitats (28). There is clearly more work to be done to resolve these questions; however, the notion that highways of gene sharing connect isolated evolutionary hot spots in the deep sea via fluid flux has intriguing evolutionary implications.

## HOW SHOULD WE STUDY MICROBIAL EVOLUTION IN THESE HABITATS?

The sequencing revolution and the accompanying wave of new bioinformatics methods have enabled researchers to peer into the genomes of microorganisms that were previously inaccessible. Most notably, recovery of metagenome-assembled genomes (MAGs) from metagenomic data enables researchers to conduct the kinds of comparative genomics analyses that were previously possible only with cultivated isolates. Through analyses of microbial genomes, we can recover the genomic “memories” of microbes living in different habitats and thus infer something about their evolutionary histories and selection pressures.

Genomics studies can be conducted both at the large scale, with a focus on microbial pangenomes and thus differences in functional capabilities across lineages, and at the fine scale, with a focus on single nucleotide or amino acid variants that provide insights into population differentiation, variation, and selection pressure (Fig. 1B). Given that variation is the material upon which selection acts, these large- and fine-scale variants provide insights into what features provide selective advantages across the subsurface.

Mapping short metagenomic reads to assembled contigs enables researchers to identify single nucleotide or single amino acid variants (29). These, in turn, can be used to identify putative signatures of selection and, in doing so, determine whether specific genes or populations are under positive or purifying selection (30), or whether they have undergone a recent clonal expansion or selective sweep (31, 32). Identification of variable gene content across closely related strains, particularly when paired with nucleotide variant analysis, can reveal how selection molds function across subsurface habitats. For example, single nucleotide variant work revealed differential selection depending on geochemical context in hydrothermal systems (23), and subsequent pangenomics work in these habitats revealed that energy and nutrient availability are among the most important selection pressures on microbial genomes in hydrothermal systems (21, 22). Similarly, *Chloroflexi* genomes recovered from marine subsurface habitats exhibit substantial metabolic flexibility and longer metabolic pathways, which may be selected in environments with limited energy and nutrient availability (33).

Future studies should focus on evolutionary dynamics across both space and time (Fig. 1C). Examination of variation across space will reveal biogeographic patterns that can reveal trends in dispersal and gene flow across subsurface habitats and ocean basins. For example, in the surface oceans, investigations have revealed differential selection in *Prochlorococcus* genomes based on phosphorus (34), while single amino acid variant analysis revealed temperature trends in SAR11 genomes (29), and yet no similar analyses have been conducted in the marine subsurface to date. In contrast, examination of genomic patterns over time can reveal how rapidly new alleles reach fixation in a population or fluctuate seasonally, as has been observed in time series observations conducted in freshwater lakes (32, 35) and in surface marine waters (36). By deploying time series studies in the marine subsurface, we can get a better handle on how quickly evolution proceeds in these habitats as well as the degree to which deterministic factors like selection or stochastic factors like genetic drift drive evolution in the subsurface. While historically time series studies in the marine subsurface have been hampered by expense and limited accessibility, new technologies such as long-term benthic landers and the Ocean Observatory Initiative (OOI) Regional Cabled Array will facilitate our ability to conduct such studies in the near future.

## WHY SHOULD WE CARE ABOUT EVOLUTION IN THE MARINE SUBSURFACE?

We cannot understand the fundamental principles that drive microbial evolution without an understanding of how evolution operates in one of the largest biospheres on Earth. Most of the foundational evolutionary microbiology studies were conducted primarily with fast-growing strains in large populations, potentially overemphasizing the speed and deterministic nature of microbial evolution. It is entirely possible that the reality of microbial evolution in vast swaths of our planet is much more stochastic and slow-paced than we previously understood. It is also possible that networks of gene sharing traveling along fluid highways in the depths of the ocean are a crucial source of evolutionary innovation. We need a more solid understanding of how microbial communities in the deep sea respond to perturbations and environmental changes in order to understand how they will respond to climate change and deep-sea mining in the short and long term. Moreover, the marine subsurface is thought to have been a crucial setting for many of the earliest stages of the evolution of life. In order to trace the grand story of life’s emergence and eventual domination of our planet, we must understand how life evolves in the massive biosphere beneath our feet and beneath our oceans. We live in an exciting time, when technological advances are enabling us to peer into the genomes of deep-sea microbes and their viruses and understand the secrets of a long-hidden world.
